# Dr. Smith's Remarks on T.Y.

**Published:** 1807-08

**Authors:** T. Smith

**Affiliations:** Edinburgh


### To Dr. BATTY.

SIR,

WHEN a disease is incurable and contagious, it is no proof of wisdom or courage to attack it; it may afford an example of temerity. If I thought the style of humour and levity an habitual and incurable disorder in the writings of your correspondent T. Y. I should exhibit my own folly in endeavouring to divert its course. That my wish to do it is strong, must be evident from volunteering my services in the most ungracious of all offices, telling a man his faults; but I am impelled by other motives, by the desire I have to amend and caution a natural and open disposition which I fancy I perceive, and from believing it cannot be difficult for a gentleman who can express his thoughts with with such freedom and sprightliness, to convey his sentiments on any subject in terms not only to arrest the attention, but claim the approbation of your readers. I beg your correspondent will not immediately fancy I am a stiff intolerant pedant, but surely, though a bruised forehead, or a broken shin, may not require much seriousness in the description, yet the remedies, whether strictly medicinal or domestic, are important acquisitions to the public, and entitled to their rank as benefactors to mankind. Every thing relating to medicine, claims the respect of the world; and if we do not promulgate its doctrines with pomp and splendor, we should be careful that its maxims are delivered with propriety and decorum. While I am recommending a little serious consideration to T. Y. befores he writes, I am as ready to praise his spirit, activity, and presence of mind, in putting to the test of experiment, two opposite modes of treatment, that certainly interest the medical world, from the indisputable authority with which they are supported. I hope, as a constant reader of your useful Journal, to be soon favoured with more of his communications, and to find I am not mistaken in the opinion I have formed of his disposition and talents.

I am, &c.

T. SMITH.

*Edinburgh, July* 6, 1807.

